# Circ-Udg Derived from Cyprinid Herpesvirus 2 Promotes Viral Replication

**DOI:** 10.1128/spectrum.00943-22

**Published:** 2022-06-30

**Authors:** Min Zhu, Yaping Dai, Xinyu Tong, Yaxin Zhang, Yang Zhou, Jiali Cheng, Yiting Jiang, Ruolin Yang, Xiangyu Wang, Guangli Cao, Renyu Xue, Xiaolong Hu, Chengliang Gong

**Affiliations:** a School of Biology and Basic Medical Sciences, Soochow University, Suzhou, China; b Agricultural Biotechnology Research Institute, Agricultural Biotechnology and Ecological Research Institute, Soochow University, Suzhou, China; c Dafeng District Aquaculture Technical Extension Station of Yancheng City, Yancheng, China; Oklahoma State University, College of Veterinary Medicine

**Keywords:** CyHV-2, circRNAs, circ-udg

## Abstract

Cyprinid herpesvirus 2 (CyHV-2) has caused great losses to the gibel carp (Carassius auratus *gibelio*) industry. Previous studies showed that certain DNA viruses can encode circular RNAs (circRNAs) to regulate virus infection, which provides new clues for the treatment of viral disease. Whether CyHV-2 can encode circRNAs is still unknown. Here, 10 CyHV-2-derived circRNAs were identified, and the function of circ-udg, a circRNA derived from the CyHV-2 uracil DNA glycosylase (*udg*) gene, was studied. Although the expression level of circ-udg was lower than that of the parental gene, *udg*, its expression level was elevated in tandem with the proliferation of CyHV-2 and *udg*. *In vitro* experiments confirmed that circ-udg could promote the proliferation of CyHV-2. Moreover, circ-udg could encode a truncated UDG protein consisting of 147-amino-acid residues (termed circ-udg-P147). Both UDG and circ-udg-P147 were found to promote CyHV-2 proliferation, but the promoting effect of circ-udg on CyHV-2 proliferation was attenuated after circ-udg lost the ability to encode circ-udg-P147. Also, circ-udg-P147 could not change the transcription level of the *udg* gene. Interestingly, the UDG protein level was increased by circ-udg-P147. These results deepen the understanding of the genetic information carried by the genome of CyHV-2 and provide a new target for the treatment of gibel carp bleeding disease caused by CyHV-2.

**IMPORTANCE** The outbreak of C. auratus *gibelio* gill hemorrhagic disease caused by CyHV-2 brought great losses to the gibel carp industry. Therefore, exploring the interaction between CyHV-2 and host and the molecular mechanism of viral infection is of great significance in preventing and treating the gibel carp gill hemorrhagic disease. Although some progress has been made in the study of CyHV-2, the mechanism of interaction between CyHV-2 and crucian carp is still unclear. In this study, we found that CyHV-2 can encode circRNA to regulate virus replication. Our study provides novel information on CyHV-2 functional genomics, a reference for research into the circRNA of other viruses, and theoretical guidance for preventing and treating gibel carp bleeding disease.

## INTRODUCTION

Cyprinid herpesvirus 2 (CyHV-2) is a double-stranded DNA virus belonging to the family *Alloherpesviridae*, order *Herpesvirales*. CyHV-2 can infect the crucian carp and its varieties, the gibel carp (Carassius auratus *gibelio*), one of China’s most important freshwater fish species. The first outbreak of C. auratus *gibelio* gill hemorrhagic disease caused by CyHV-2 brought great losses to the gibel carp industry in Yancheng City, Jiangsu Province, China (1). Therefore, exploring the interaction between CyHV-2 and host and the molecular mechanism of viral infection is of great significance in preventing and treating gibel carp gill hemorrhagic disease. Current research efforts on CyHV-2 mainly focus on sequencing and functional annotation of the genome of CyHV-2 (2–6), the response of the gibel carp to CyHV-2 at the whole-transcriptome level, and the development of anti-CyHV-2 vaccines (7). Bioinformatics analysis indicated 154 predicted open reading frames (ORFs) in the genome of CyHV-2, but the functions of most genes are unknown (2). A previous study demonstrated that ORF104 of CyHV-2 encodes a protein kinase, which can activate the p38-MAPK signaling pathway during CyHV-2 infection, and inhibiting this pathway could reduce the lethality of CyHV-2 (8). Although some progress has been made in the study of CyHV-2, the mechanism of interaction between CyHV-2 and crucian carp is still unclear.

Circular RNAs (circRNAs) are a class of closed circular molecules without a 5′ terminal cap and 3′ terminal poly(A) tail. These circRNAs have strong stability and are not degraded by RNA exonuclease (9). Studies have shown that circRNAs can function as a microRNA (miRNA) “sponge,” interact with RNA binding proteins (RBPs), participate in various biological functions such as gene transcription and splicing regulation, and play an important role in many processes such as cell proliferation, differentiation, survival, and senescence (10–15). Studies have also shown that circRNAs can translate peptides or proteins in a cap-independent manner (14–17). However, most of these studies on the functions of circRNAs focus on endogenous circRNAs produced by cellular transcripts. Recent studies showed that in addition to the circRNAs from cell transcripts, the transcripts of some viruses, such as the Kaposi's sarcoma-associated herpesvirus (KSHV), Epstein-Barr virus (EBV), hepatitis B virus (HBV), human papillomavirus (HPV), and rhesus macaque lymphocryptovirus (rLCV), can also form circRNAs, which play important roles in regulating viral proliferation and infection process (14, 15, 17–22).

In this study, using circRNAome sequencing (circRNA-Seq), divergent PCR, and Sanger sequencing, we demonstrate that CyHV-2 could generate circRNAs. The ORF98 of CyHV-2 encodes uracil DNA glycosylase (UDG), and no functional studies have been conducted on the *udg* gene. In the functional gene annotation of CyHV-2, it is considered that *udg* is involved in the repair of the viral DNA (see GenBank accession no. NC_019495). Here, we identified that a circRNA was generated from *udg* genes, termed circ-udg. It was found that the expression level of circ-udg increased with the proliferation of CyHV-2 and that circ-udg can promote CyHV-2 proliferation. Furthermore, circ-udg was found to encode a truncated UDG protein consisting of 147-amino-acid (aa) residues (herein termed circ-udg-P147). Functionally, upregulation of the expression level of circ-udg-P147 expression and UDG promoted virus proliferation *in vitro*. When the start codon of the circ-udg-P147 protein (encoded in circ-udg) is mutated, its promoting effect on virus proliferation is attenuated. Our study provides novel information on CyHV-2 functional genomics, a reference for research into the circRNA of other viruses, and theoretical guidance for preventing and treating gibel carp bleeding disease.

## RESULTS

### Overview of circRNAs derived from CyHV-2.

Here, to characterize the circRNAs in the Carassius auratus *gibelio* after CyHV-2 infection, the clean reads from the RNase R-resistant RNA circRNAome sequencing data (23) were aligned with the CyHV-2 genome. CIRI2 and Find_circ (the first software to predict circRNAs using high-throughput sequencing data) software were utilized to predict the circRNAs. In total, 10 potential CyHV-2 circRNAs with head-to-tail joining were identified in CyHV-2-infected gibel carp; 5 of the 10 circRNAs were distributed in the positive chain of the CyHV-2 genome, while the remaining 5 were distributed in the negative chain. The size of circRNAs ranged from 280 to 40,679 nucleotides (nt), and the back-splicing signal of circRNA does not follow the GU-AG rules (Table 1). Moreover, divergent PCR and Sanger sequencing were conducted to verify the circRNAome sequencing results. Sequences of the junction site obtained through Sanger sequencing were consistent with those obtained by circRNAome sequencing (see Fig. S1 in the supplemental material).

**TABLE 1:** Primers used in this study

Primer name	Sequence	
	Forward (5′→3′)	Reverse (5′→3′)
CyHV2V-circ1	TATGGCTGTGCCTCAACGAG	TGCGATATCCAAGAGTCGGC
CyHV2V-circ2	GTCACCACCTGTATGCCCTT	GTCGGGCTTGTAGATGTCGT
CyHV2V-circ3	AACGCACGGAGAGAGGGTA	ATGACCGACTCTGGTTCGAG
CyHV2V-circ4	ATCCCGAAGAGAACGTGGAAG	GGCTTCGTCTTCGTCGTGTTA
CyHV2V-circ5	CTTGGACAGATCCCCGAGTC	ACAAAGAGGTGGTCAACGGT
CyHV2V-circ6	TGCGTCTTCACAA CGGACTA	AGTCAATCCGACGCTCAGAC
CyHV2V-circ7	TGCCAAACTCAGACAACGCT	AGGTCAATGTAGGTGCCAGG
CyHV2V-circ8	CCCAAGAAGAAGCAGCCAAC	ACCGTTACCAGTCCAGCATT
CyHV-2V-circ-udg	TGCGTCTTCACAA CGGACTA	AGTCAATCCGACGCTCAGAC
circ-udg-P147	GGATCCATGGGTTTTGGGACTACGG	CTCGAGTCAATCTATAATCATATTGGCGTCTTTG
pmCherry-C1-circ-udg-P147	GAATTCATGGGT TTTGGGACTACGG	GGATCCTCAATCTATAATCATATTGGCGTCTTTG
pZsGreen1-C1-UDG	GAATTCATGTCGAAGAGGAAGGCTG	GGATCCTCAATCTATAATCATATTGGCGTC
T7-circ-udg	TAATACGACTCACTATAGGGTTTCAACGGTATGGGTTTTG	AATCGGTAATTAGCCTTGAGAG
T7-circ-GFP	TAATACGACTCACTATAGGCAAGCTGACCCTGAAGTTC	GTAGTTGTACTCCAGCTTGTG
T7-circ-udgmut	TAATACGACTCACTATAGGGTTTCAACGGTTAGGGTTTTG	AATCGGTAATTAGCCTTGAGAG
circ-udg	CTCTCAAGGCTAATTACCGATTGT	ACACAAGACACCCTGCTCTG
40S rRNA	CCGTGGGTGACATCGTTACA	TCAGGACATTGAACCTCACTGTCT
ORF72	TCCACCGACTCAAAAACGGT	TGACCGCCATAAGCGTTGTA
ORF71 (helicase)	GGGTGAGGACTTGCGAAGAG	CGCTCGTCCGGGTTCTGCACG
udg	CCGTCAGAGGTACAGAGGAGA	ACCTTCGTCCTCGTTGTTGT
Biotin-circ-udg	AGAGTTCCGATT AATGG	CTAACAAA GTTGCCATACCCAAAAC

### Identification of circ-udg.

**FIG 1:**
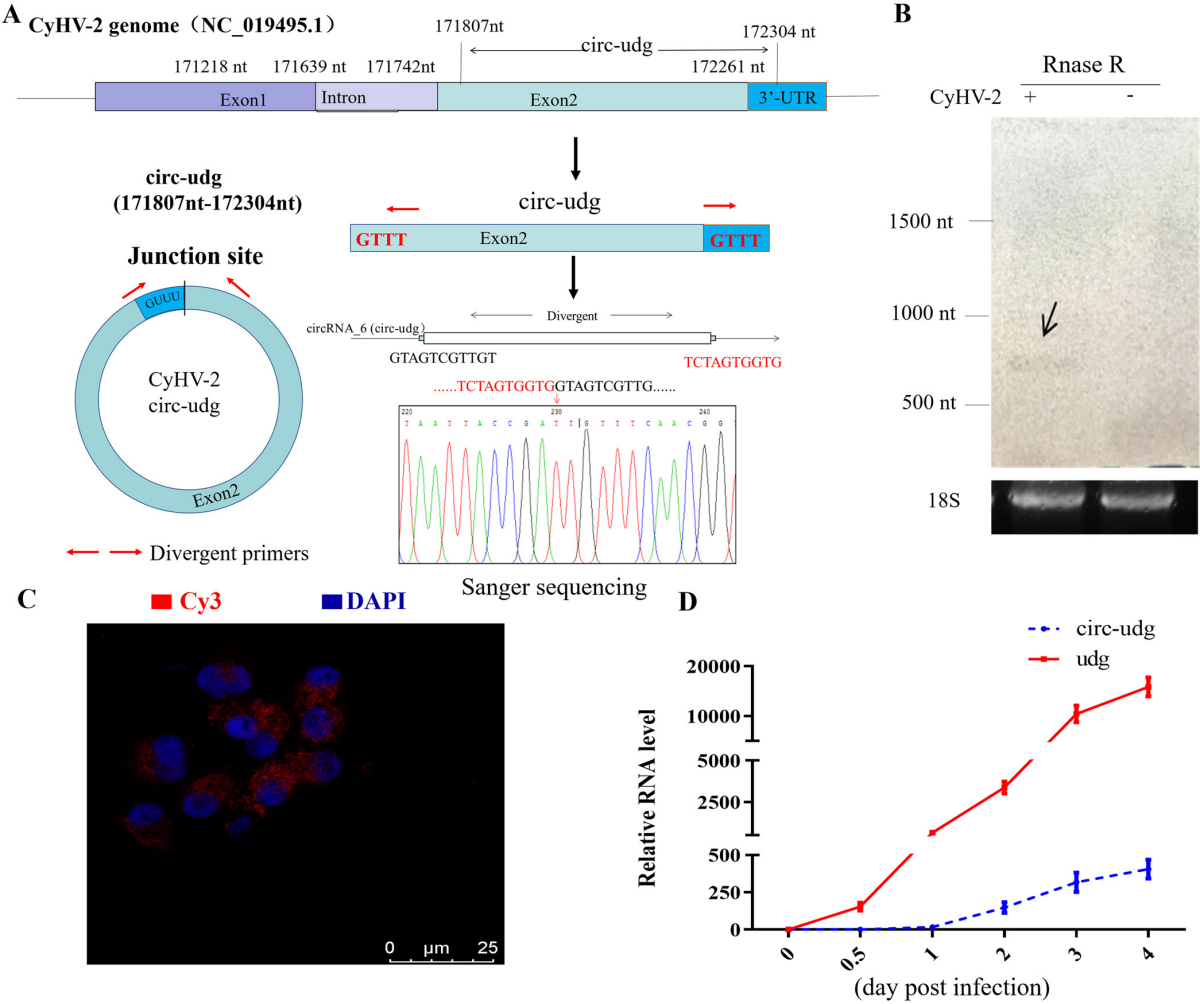
Validation of circ-udg. (A) Sketch of the genomic locus of circ-udg in CyHV-2 *udg* gene. Red arrows represent divergent primers. (B) Expression of circ-udg (GenBank accession no. NC_019495; 171,807 nt to 172,304 nt) was validated by divergent PCR followed by sanger sequencing. Red arrows represent divergent primers, which are used to amplify the genome region of circ-udg-containing junction site. (C) circ-udg was validated by Northern blotting. 18S represents 18S rRNA as internal control. (D) Representative image of RNA fluorescence *in situ* hybridization for circ-udg in CyHV-2-infected EPC cells. Cell nuclei were counterstained with 4,6-diamidino-2-phenylindole (DAPI). (E) mRNA of udg and circ-udg expression pattern in CyHV-2-infected EPC cells. CyHV-2-infected EPC cells were collected at 0, 0.5, 1,2, 3, and 4 days postinfection, expression levels of *udg* and circ-udg were determined by real-time PCR, and the 40S rRNA gene was used as an internal control.

Studies have shown that herpesviruses can encode UDG to participate in virus replication (24). The *udg* gene was encoded by CyHV-2 (GenBank accession no. NC_019495) ORF98 (located in the 171,218- to 172,348-nt region) (25). Here, circRNA-Seq identified a circRNA circ-udg whose sequence corresponds to the 171,807- to 172,304-nt region on the genome of CyHV-2 (Fig. 1A). Furtherly, circ-udg was identified by PCR with divergent primers and convergent primers, and sequences flanking the junction site and complete sequences of circ-udg obtained through Sanger sequencing of the PCR products were consistent with those obtained via high-throughput sequencing (Fig. 1A). Northern blotting also detected a specific band in a CyHV-2-infected fish kidney with an oligonucleotide probe targeting the circ-udg junction site (Fig. 1B). *In situ* hybridization showed that circ-udg was mainly localized in the cytoplasm (Fig. 1C).

Furthermore, the relative expression levels of circ-udg and its parental gene, *udg*, were determined by real-time PCR in CyHV-2-infected epithelioma papulosum cyprini (EPC) cells. The results showed that the expression of *udg* was detectable at 0.5 days postinfection (dpi) and that the expression level of *udg* increased sharply with the infection of CyHV-2. The expression of circ-udg was detected at 1 dpi, and its expression level also increased with CyHV-2 infection; however, the expression level of circ-udg was generally lower than that of *udg* (Fig. 1D).

### circ-udg promoting viral proliferation.

**FIG 2:**
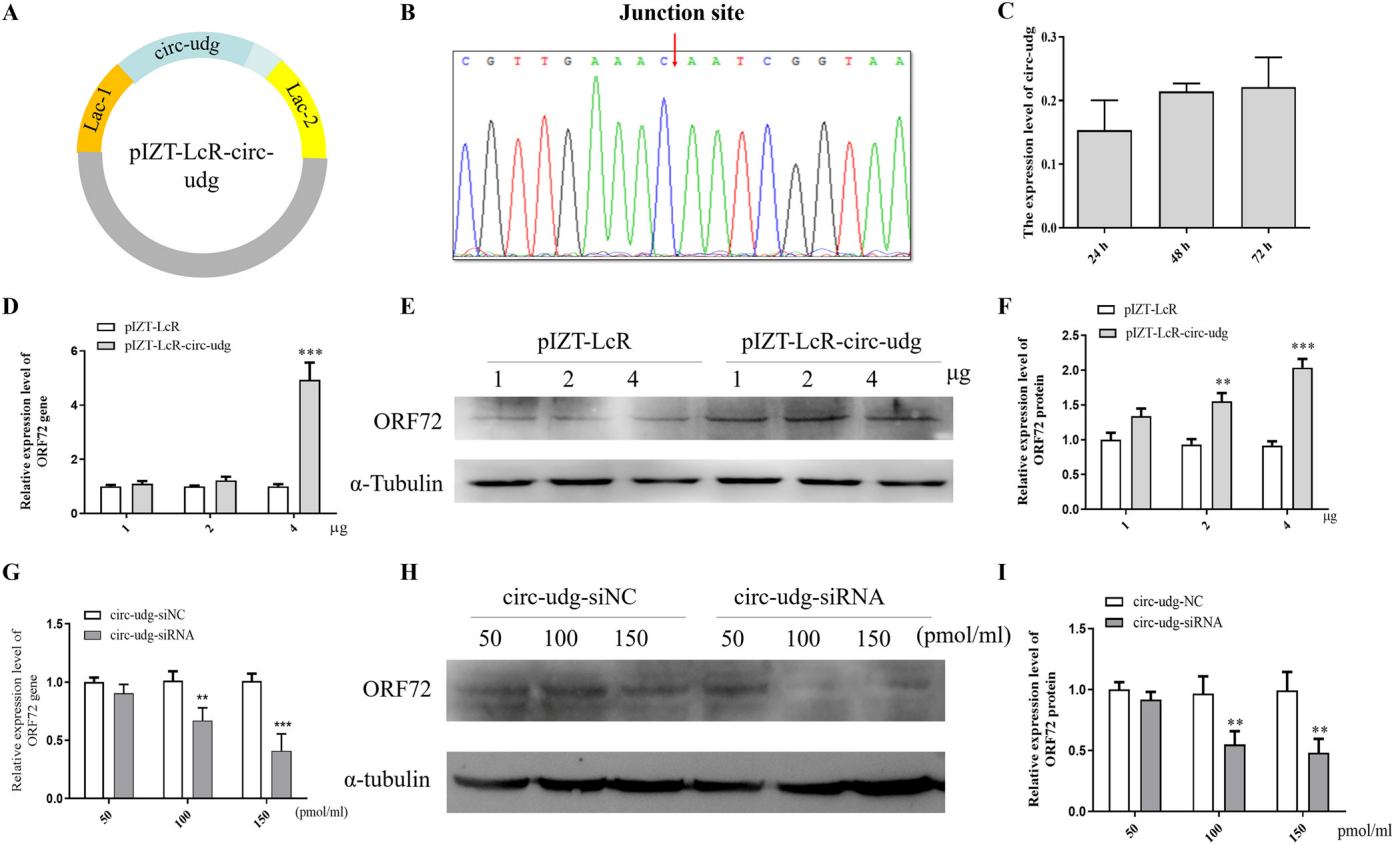
circ-udg promotes CyHV-2 replication. (A) Structural diagram of pIZT-LcR-circ-udg vector. (B) Sanger sequencing of the junction sites of circ-udg expressed by pIZT-LcR-circ-udg in EPC cells. (C) Expression of circ-udg in the transfected EPC cells of pIZT-LcR-circ-udg. We transfected 2 μg pIZT-LcR-circ-udg into 1 × 10^6^ cells, and the cells were collected after 24 h, 48 h, and 72 h, respectively. Then, RNA was extracted and reverse transcribed to cDNA with random primers. The expression levels of circ-udg were detected by real-time PCR, with the 40s rRNA gene as an internal control. (**, *P < *0.01; *n* = 2). (D and E) Effect of overexpressing circ-udg on CyHV-2 *ORF72* gene at mRNA and protein levels. We transfected 1 × 10^6^ cells with 1, 2, or 4 μg of pIZT-LcR-circ-udg or pIZT-LcR vector, and 48 h later, the transfected cells were infected by 100 μL CyHV-2 (1 × 10^5^ copies/μL). (D) RNA was extracted at 48 h postinfection and reverse transcribed to cDNA with random primers. The expression levels of *ORF72* were determined by real-time PCR, with the 40S rRNA gene as an internal control. (E) Proteins were collected for Western blotting. The primary antibody was CyHV-2 ORF72 antibody (1:800 dilution). The secondary antibody was HRP-labeled goat anti-mouse antibody (1:5,000 dilution), and the internal control was α-tubulin. (F) Grayscale scanning analysis of Western blotting signal bands (*, *P* < 0.05; **, *P < *0.01; ***, *P < *0.001; *n* = 3). (G and H) Effect of silencing circ-udg on CyHV-2 *ORF72* gene at mRNA and protein levels. The cells (1 × 10^6^) were infected by 200-μL CyHV-2 crude extracts (1 × 10^5^ copies/μL). Precisely 50, 100, and 150 pmol of circ-udg-siRNA2 were transfected into the cells at CyHV-2 48 h postinfection, respectively. After 48 h, RNA or protein was extracted, and expression of the *ORF72* gene was detected by real-time PCR and Western blotting at mRNA and protein levels. (I) Grayscale scanning analysis of Western blotting signal bands.

To study the function of circ-udg, we cloned the linear DNA sequence of circ-udg into a circRNA expression vector, pIZT-lcR (26), to construct the circ-udg expression vector pIZT-LcR-circ-udg (Fig. 2A). Following transfection of pIZT-lcR-circ-udg into EPC cells, circ-udg was detectable by divergent PCR, and Sanger sequencing results showed that the flanking sequences of the circ-udg junction site were as expected (Fig. 2B), indicating that the pIZT-lcR-circ-udg could correctly express circ-udg in cells. Moreover, the expression level of circ-udg in the transfected cells with pIZT-lcR-circ-udg was determined by real-time PCR at 24, 48, and 72 h posttransfection. Real-time PCR results showed that the expression level of circ-udg increased in a time-dependent manner after transfection (Fig. 2C). Functionally, in the transfected cells with pIZT-LcR-circ-udg, the expression level of the *ORF72* gene of CyHV-2 was detected by real-time PCR and Western blotting. Compared with the transfected cells with pIZT-LcR, the transcription level of *ORF72* genes was significantly higher in the transfected cells with 4 μg of pIZT-LcR-circ-udg (Fig. 2D), while ORF72 protein expression levels were upregulated 1.5 and 2.2 times in the transfected cells with 2 and 4 μg of pIZT-LcR-circ-udg, respectively (Fig. 2E and F).

Additionally, specific small interfering RNAs (circ-siRNAs) targeting the circ-udg junction site sequences were transfected into CyHV-2-infected cells at various final concentrations of 50, 100, and 150 pmol/mL, transfecting cells with the corresponding dose of siRNA-NC as controls. The relative expression levels of the CyHV-2 *ORF72* gene and ORF72 protein were determined by real-time PCR and Western blotting, respectively. Compared to the control group, the transcription of the *ORF72* gene was downregulated 0.7-fold and 0.4-fold (Fig. 2G), while the expression of the ORF72 protein was downregulated 0.7-fold and 0.6-fold in groups transfected with 100 and 150 pmol of circ-siRNA, respectively (Fig. 2H and I). Altogether, these results suggest that circ-udg could promote the proliferation of CyHV-2.

### circ-udg encodes protein circ-udg-P147.

**FIG 3:**
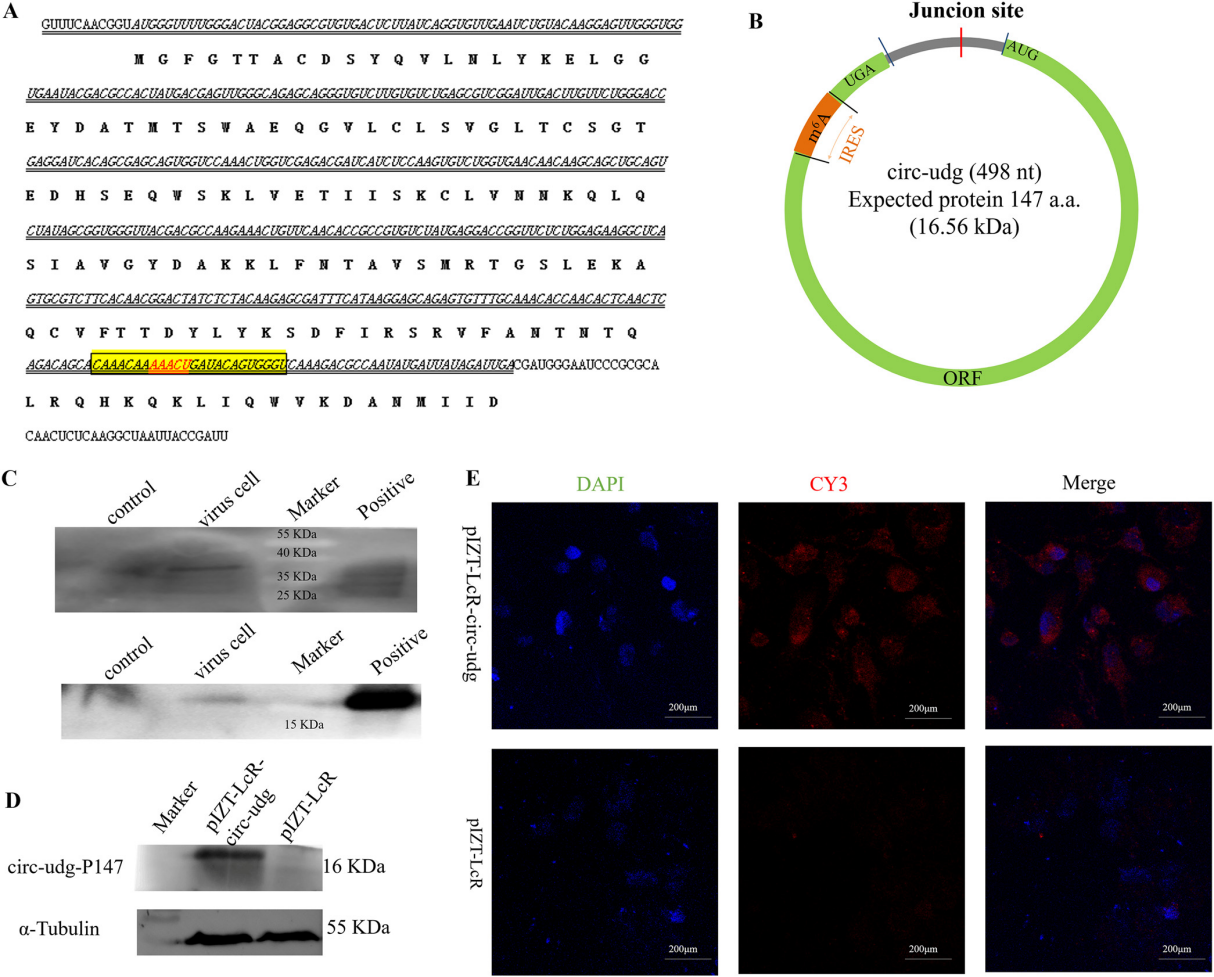
circ-udg encodes the protein circ-udg-P147. (A) The circ-udg sequence, the IRES sequence, the putative m6A site, and encoded amino acid sequences. Sequences in italics represents the putative ORF, boldface represents the amino acid sequence of circ-udg-P147 encoded by circ-udg, and the underlined sequences represents the specific amino acid sequence of circ-udg-P147. Red font represents the m6A site. (B) Structure of circ-udg. (C) circ-udg-P147 and UDG were detected by Western blotting in CyHV-2-infected EPC cells. (D) circ-udg-P147 was detected by Western blotting in the pIZT-LcR-circ-udg-transfected cells. EPC cells (1 × 10^6^) were transfected with 8 μg pIZT-LcR-circ-udg. After 6 days, cellular proteins were collected for Western blotting. (E) Cell immunofluorescence assay of circ-udg-P147 in the pIZT-LcR-circ-udg-transfected cells. The pIZT-LcR-circ-udg-transfected EPC cells (1 × 10^4^) were subjected to immunofluorescence with anti-circ-udg-P147 antibody. The nucleus was stained with DAPI.

Bioinformatics analysis revealed the presence of an internal ribosome entry site (IRES)-like sequence (CAAACAAAAACTGATACAGTGGGT), N^6^-methyladenine (m6A) methylation motif, and an ORF potentially encoding a protein (circ-udg-P147) with 147-amino-acid residues in the circ-udg (Fig. 3A and B). Alignment analysis showed that the amino acid sequence of circ-udg-P147 was consistent with the 147 amino acid residues at the C-terminal CyHV-2 UDG protein. To verify circ-udg-P147 encoded by circ-udg, a mouse anti-circ-udg-P147 polyclonal antibody was prepared. This antibody could successfully recognize the recombinant protein fused with a His tag (data not shown). Then, the proteins extracted from CyHV-2-infected EPC cells were used for immunoprecipitation with an anti-circ-udg-P147 antibody. The precipitated complexes were used for Western blot detection. A 37-kDa signal band representing UDG (Fig. 3C) and a 16-kDa signal band representing circ-udg-P147 could be found, while no specific bands were observed in the uninfected EPC cells (Fig. 3C), suggesting that circ-udg-P147 existed in CyHV-2-infected EPC cells. Moreover, a specific band of about 16 kDa was detected in EPC cells transfected with circ-udg expression vector pIZT-lcR-circ-udg, while no bands were observed in cells transfected with vector pIZT-lcR (Fig. 3D). The immunofluorescence results also showed a red fluorescence representing circ-udg-P147 in EPC cells transfected with pIZT-lcR-circ-udg, but not in the cells transfected with pIZT-lcR (Fig. 3E). Collectively, these results suggest that circ-udg can encode circ-udg-P147 protein.

### circ-udg encodes circ-udg-P174 through IRES.

**FIG 4:**
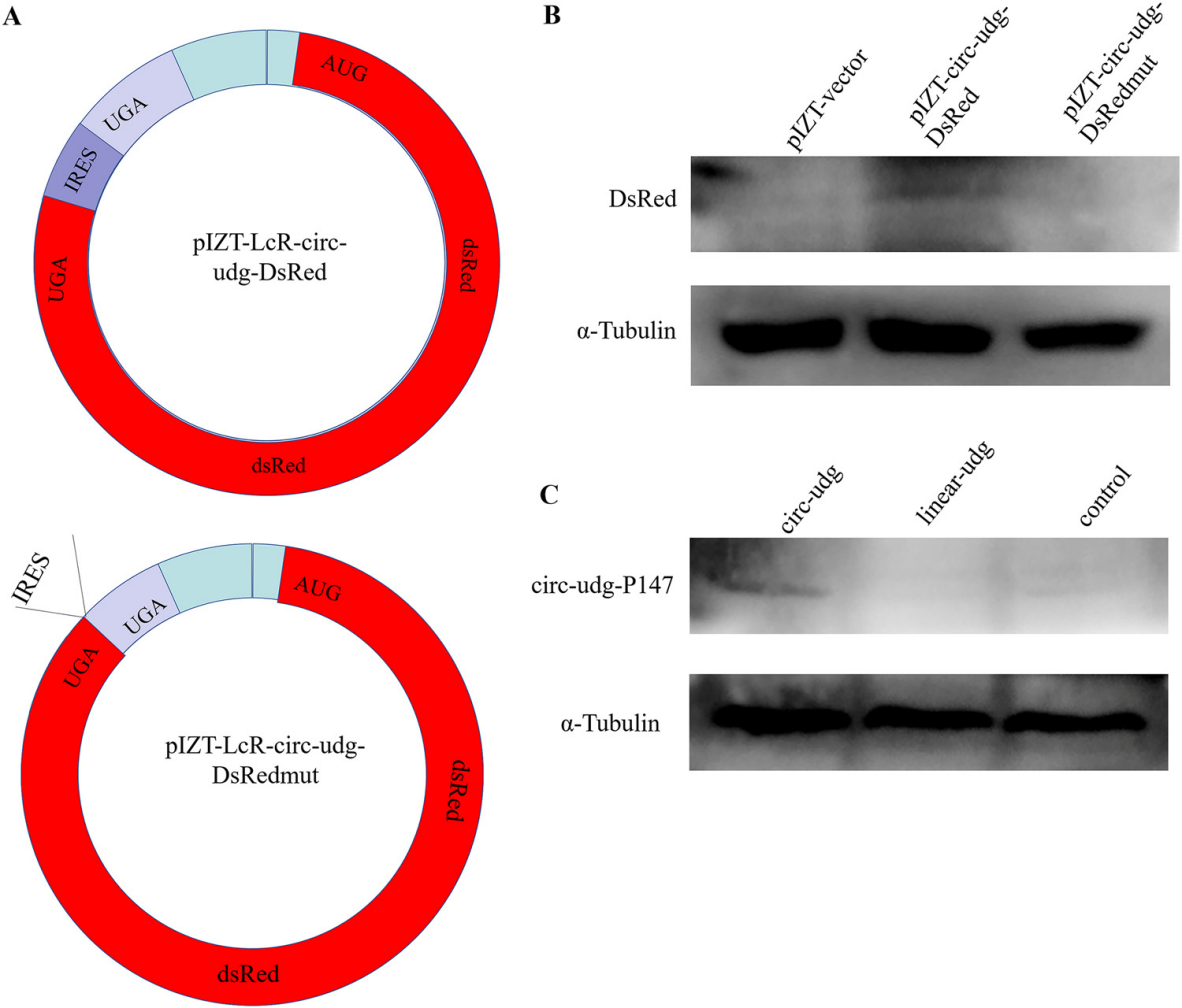
circ-udg encode circ-udg-P147 via internal ribosome entry site (IRES). (A) Schematic diagram of plasmids pIZT-LcR-circ-udg-DsRed and pIZT-LcR-circ-udg-DsRedmut. To construct pIZT-LcR-circ-udg-DsRed, the ORF of circ-udg-P147 in pIZT-LcR-circ-udg was replaced by the coding sequence of DsRed. pIZT-LcR-circ-udg-DsRedmut was constructed by deleting the IRES in pIZT-LcR-circ-udg-DsRed. (B) DsRed was detected by Western blotting in pIZT-LcR-circ-udg-DsRed- or pIZT-LcR-circ-udg-DsRedmut-transfected cells. We transfected 1 × 10^6^ EPC cells with 8 μg pIZT-LcR-circ-udg-DsRed, pIZT-LcR-circ-udg-DsRedmut, and pIZT-LcR. After 6 days, cellular proteins were collected for Western blotting with anti-DsRed antibody. (C) circ-udg-P147 was detected by Western blotting in the cells transfected with *in vitro*-synthesized circ-udg. The linear form of circ-udg that was synthesized *in vitro* was used as control.

To understand the mechanism of circ-udg translating circ-udg-P147, we constructed a pIZT-lcR-circ-udg-dsRED vector by using DsRed cDNA sequence to replace circ-udg-P147 ORF in pIZT-lcR-circ-udg. Meanwhile, the vector pIZT-LcR-circ-udg-dsREDmut with the IRES deletion was constructed (Fig. 4A). Western blotting showed that DsRed protein could be detected in the cells transfected with pIZT-lcR-circ-udg-dsRED, but not in the cells transfected with pIZT-LcR-circ-udg-dsREDmut (Fig. 4B), indicating that the translation initiation of circ-udg-P147 was mediated by IRES. Bioinformatics analysis showed that the predicted IRES also contains an m6A methylation motif. Previous studies have shown that the circRNA synthesized *in vitro* is not methylated (27). Therefore, if the translation of circ-udg-P47 is initiated by m6A, the circ-udg-P47 protein cannot be detected in the cells transfected with circ-udg synthesized *in vitro*. To explore whether the translation of circ-udg-P47 is IRES or m6A dependent, the linearized circ-udg and circ-udg generated by transcription *in vitro* were transfected into EPC cells, and then the circ-udg-P147 protein was detected by Western blotting. It was found that circ-udg-P147 protein could be detected only in cells transfected with circ-udg (Fig. 4C), indicating that circ-udg initiated the translation of circ-udg-P147 protein through IRES, and the linearized circ-udg could not encode the protein.

### circ-udg-P147 promotes viral proliferation.

**FIG 5:**
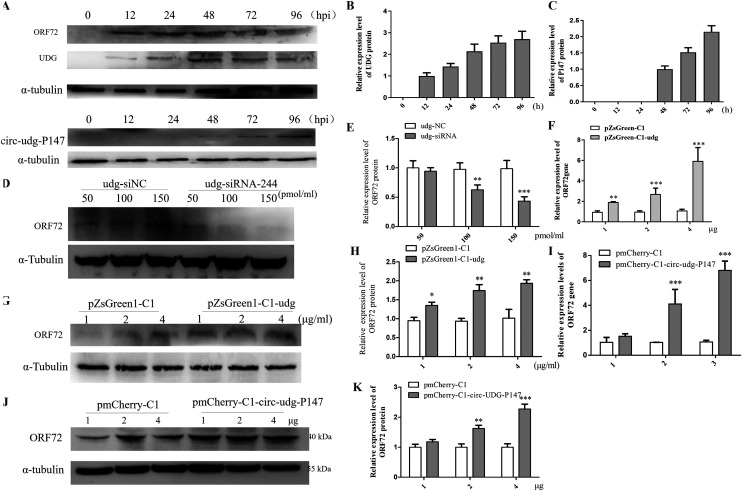
circ-udg-P147 promotes CyHV-2 replication. (A) UDG protein and circ-udg-P147 protein expression phase during the progression of CyHV-2 infection. We infected 1 × 10^6^ EPC cells with 200 μL CyHV-2 (2 × 10^6^copies/μL), and the cells were collected at 0, 0.5, 1, 2, 3, and 4 days postinfection. The levels of UDG protein and circ-udg-P147 were determined by Western blotting. (B and C) Grayscale scanning analysis of Western blotting signal bands. (D) Effect of silencing udg on the level of ORF72 protein. Precisely, 200 μL of CyHV-2 (1 × 10^6^copies/μL) was used to infect the cells (1 × 10^6^), and different doses of udg-siRNA (50, 100, and 150 pmol) were transfected into the cells at 48 hpi. After 48 h, the protein was extracted for Western blotting with anti-ORF72 antibody. (E) Grayscale scanning analysis of Western blotting signal bands. (F) Effect of overexpressing udg on the expression of *ORF72* at mRNA level. We transfected 1 × 10^6^ cells with 1, 2, and 4 μg pZsGreen1-C1-udg, respectively. After 48 h, the cells were infected with 200 μL CyHV-2 (1 × 10^6^copies/μL), and the expression of the *ORF72* gene was detected by real-time PCR at 48 hpi. (G) Effect of overexpressing udg on the expression of ORF72 protein. The treatment of cells was the same as those described in panel F. The protein was extracted for Western blotting with anti-ORF72 antibody. (H) Grayscale scanning analysis of Western blotting signal bands. (I) Effect of overexpressing circ-udg-P147 on the expression of *ORF72* at mRNA level. We transfected 1 × 10^6^ cells with 1, 2, and 4 μg pmCherry-C1-circ-udg-P147, respectively. After 48 h, the cells were infected by 200 μL CyHV-2 (1 × 10^6^copies/μL), and the expression of *ORF72* gene was detected by real-time PCR at 48 hpi. (J) Effect of overexpressing circ-udg-P147 on the expression of ORF72 protein. The treatment of cells was the same as those described in panel I. The protein was extracted for Western blotting with anti-ORF72 antibody. (K) Grayscale scanning analysis of Western blotting signal bands.

In order to explore the potential function of circ-udg-P147, we detected the expression patterns of circ-udg-P147 and UDG during CyHV-2 infection by Western blotting. The results showed that UDG were detectable at 12 h postinfection (hpi) with CyHV-2 (Fig. 5A and B), whereas the circ-udg-P147 protein was detectable at 48 hpi (Fig. 5A and C). The expression levels of circ-udg-P147 and UDG all increased with CyHV-2 infection, suggesting circ-udg-P147 plays potential regulatory roles during CyHV-2 infection. Because circ-udg-P147 is a truncated UDG protein and is in complete agreement with the sequence of the CyHV-2 UDG C-terminal 147 amino acids, we first investigated the effect of regulating udg genes on CyHV-2 infection. It was found that when the udg gene was silenced by 100 and 150 pmol of udg-siRNA, the CyHV-2 ORF72 protein was downregulated approximately 0.7-fold and 0.4-fold, respectively (Fig. 5D and E). Furthermore, in the CyHV-2-infected cells transfected with 1, 2, and 4 μg of UDG overexpression plasmid pZsGreen1-C1-udg, the transcript levels of ORF72 gene in the cells were upregulated about 2-, 3-, and 5-fold (Fig. 5F), while the levels of ORF72 protein were upregulated about 1.36-, 1.8-, and 2.0-fold respectively (Fig. 5G and H). These results suggest that UDG contributes to CyHV-2 infection.

Next, the function of circ-udg-P147 on CyHV-2 proliferation was explored. The EPC cells were transiently transfected with 2 or 4 μg of circ-udg-P147 expression plasmid pmCherry-C1-circ-udg-P147, followed by infection with CyHV-2. In parallel, transfection of equal doses of pmCherry-C1 plasmid was used as a control. Compared with their parental cells, in cells transfected with 2 and 4 μg of pmCherry-C1-circ-udg-P147, the expression of the CyHV-2 *ORF72* gene increased at both the mRNA and protein levels in a dose-dependent manner (Fig. 5I to K), suggesting that circ-udg-P147 plays an important role in CyHV-2 infection.

### Decreased ability of circ-udg to regulate viral gene expression upon loss of ability to encode circ-udg-P147.

**FIG 6:**
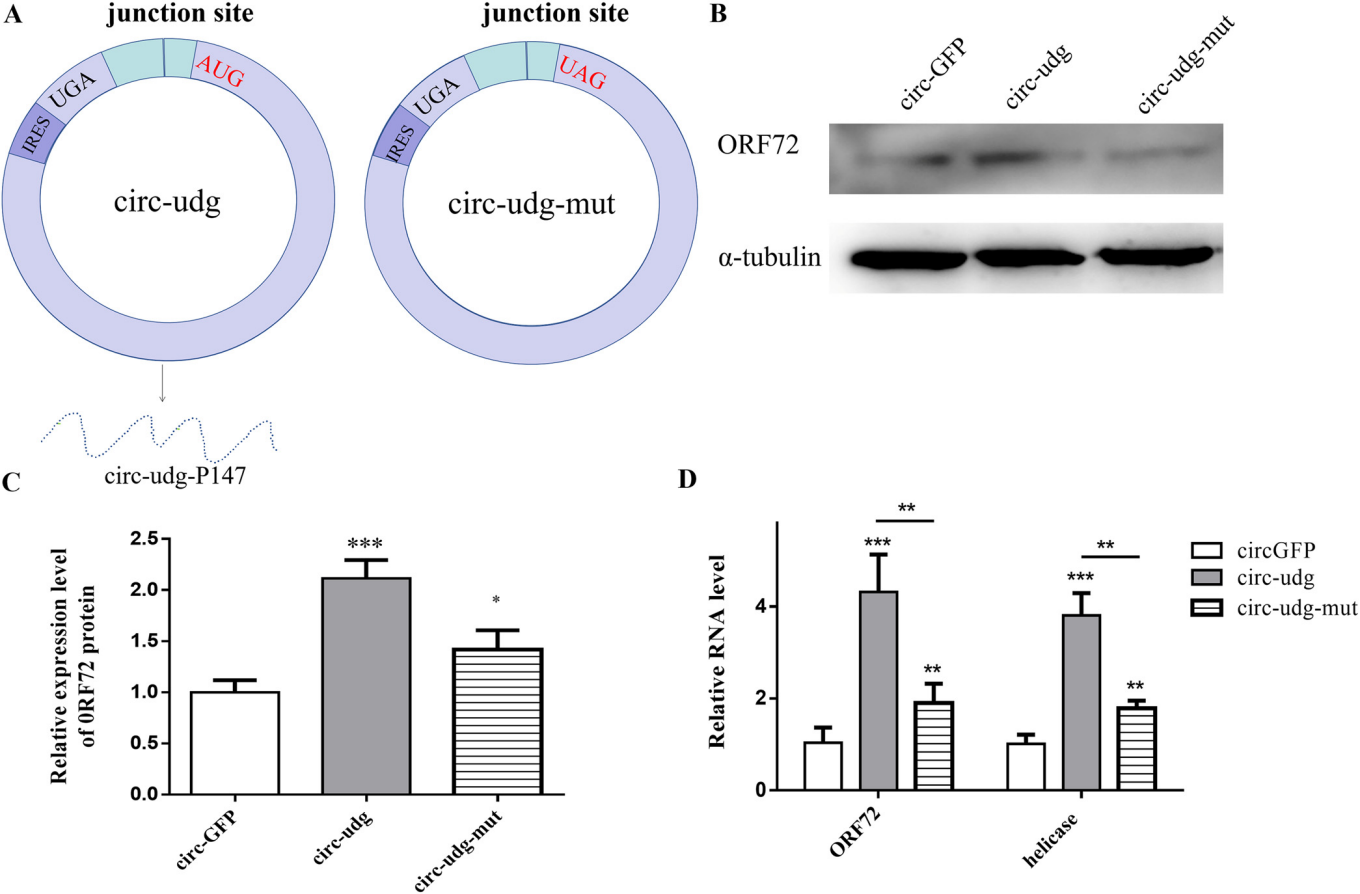
Loss of coding capacity of circ-udg decreased the ability of circ-udg to regulate viral proliferation. (A) Structure diagram of circ-udg and circ-udg-mut. circ-udg could express circ-udg-P147, but in circ-udg-mut, the start codon of ORF of circ-udg-P147 was mutated to the stop codon, which could not express circ-udg-P147 protein. (B) Effect of circ-udg losing the ability to encode circ-udg-P147 on virus genes. We transfected 4 μg of circ-GFP, circ-udg, and circ-udg-mut into 1 × 10^6^ EPC cells. After 48 h, 200 μL CyHV-2 (1 × 10^6^ copies/μL) was inoculated. The relative expression of the *ORF72* gene and helicase gene were detected by real-time PCR at 48 hpi. (C) Effect of circ-udg losing the ability to encode circ-udg-P147 on virus protein. The treatment of cells was the same as those described in panel B. The protein was extracted for Western blotting with anti-ORF72 antibody. (D) Grayscale scanning analysis of Western blotting signal bands.

The above findings revealed that both circ-udg and circ-udg-P147 increased the expression of the CyHV-2 *ORF72* gene. Next, we investigated whether circ-udg functions directly in the form of its RNA molecules or indirectly by encoding the circ-udg-P147 protein. To achieve this, we mutated the start codon of the circ-udg-P147 ORF to a stop codon to explore how circ-udg functions (Fig. 6A). Real-time PCR results showed that the capability of circ-udg to upregulate CyHV-2 *ORF72* and helicase genes decreased when the protein-coding function of circ-udg was lost (Fig. 6B). Similarly, Western blotting results showed that the mutated circ-udg was less able to upregulate CyHV-2 ORF72 protein levels than the functional circ-udg (Fig. 6C and D). However, the mutated circ-udg also promoted CyHV-2 replication, suggesting that circ-udg functions directly in the form of its RNA molecules and indirectly by encoding the circ-udg-P147 protein.

### Circ-udg-p147 could increase the protein level of UDG.

**FIG 7:**
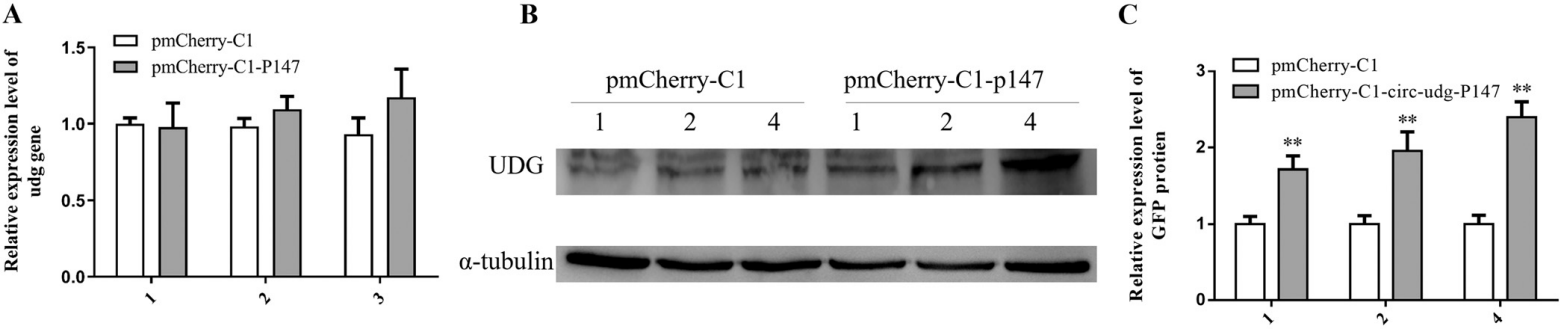
circ-udg-P147 can increase the level of UDG protein. (A) Effect of circ-udg-P147 on the transcription level of *udg* gene. Different doses of circ-udg-P147 overexpression plasmid pmCherry-C1-circ-udg-P147 (1, 2, and 4 μg) were cotransfected with 4 μg UDG overexpression plasmid pZsGreen1-C1-udg into EPC cells (1 × 10^6^), respectively. Different doses of plasmid pmCherry-C1-circ-udg-P147 were cotransfected with 4 μg plasmid pZsGreen1-C1 as control. After 96 h, RNA was extracted, and the relative expression level of *udg* gene at mRNA level was detected by real-time PCR. (B) Effect of circ-udg-P147 on the level of UDG protein. Cells were treated as described above. Cellular proteins were extracted and then detected by Western blotting. Primary antibody was anti-green fluorescent protein (GFP) antibody (1:2,000 dilution), the secondary antibody was HRP-labeled goat anti-mouse antibody (1:5,000 dilution), and the internal reference was α-tubulin. (C) Grayscale scanning analysis of Western blotting signal bands (*, *P < *0.05; **, 0.05* < P < *0.01; *n* = 3).

It has been reported that the protein encoded by the circRNA can increase the level of the protein encoded by its parental gene by protecting the parental protein from degradation (28). Therefore, we investigated whether circ-udg-P147 has a similar function. To this end, we cotransfected cells with pmCherry-C1-circ-udg-P147 and pZsGreen1-C1-udg expression plasmids and then performed real-time PCR and Western blotting to detect the effect of circ-udg-P147 on *udg* gene expression at mRNA and protein levels. Compared to the control, the mRNA level of *udg* did not change in a transfected pmCherry-C1-circ-udg-P147 dose-dependent manner (Fig. 7A), but the protein level of UDG increased in a dose-dependent manner (Fig. 7B and C). However, whether circ-udg-P147 increases the level of UDG protein by protecting UDG from degradation requires further investigation.

## DISCUSSION

CircRNA has been found in many organisms, but there are few reports about viruses coding circRNAs. EBV and KSHV, which belong to the family of *Herpesviridae*, were found to generate circRNA by high-throughput sequencing combined with divergent PCR and Sanger sequencing (21, 22). CyHV-2 belongs to the genus *Cyprinivirus* of *Herpesviridae*. Here, a total of 10 CyHV-2-derived circRNAs were identified by circRNA-Seq. Furthermore, the flanking sequences of the junction site of circRNAs obtained by divergent PCR and Sanger sequencing were consistent with the high-throughput sequencing results, indicating that CyHV-2 can indeed generate circRNAs. In eukaryotic cells, endogenous circRNAs originate from linear transcripts of parental genes by back splicing with the canonical splicing signal GU-AG (29). Previous findings have shown that some of the genes of large DNA viruses have introns. Thus, primary transcripts of these genes need to be processed to form mature RNAs, thereby leading to the formation of intronic circRNAs and exon-intron circRNAs in some viruses (18). CyHV-2 is a large DNA virus with 290 kb of genomic DNA. In this study, we found that CyHV-2-encoded circRNAs can also be classified into intergenic circRNAs, exonic circRNAs, and exon-intron circRNAs, but their splicing signals are not identical to those of endogenous circRNAs (GU-AG), which may be due to the splicing signals of some mRNAs of viruses that do not follow the GT-AG rule (30).

Studies have shown that in some organisms, the 5′- and 3′-end split sites of circRNAs tend to have short direct repeats, which are important for circRNA formation, and only one of these repeats is retained in the formed circRNA (31). Similar short direct repeats were found on the 5′- and 3′-end split sites of circ-udg. There was a GUUUU sequence downstream of the 5′- and 3′-end split sites of circ-udg, and only one GUUUU sequence was retained in the formed circ-udg. Therefore, subsequent studies are required to explore the role of GUUUU repeats in the formation of circ-udg.

An increasing number of studies have shown that in addition to mRNA, some viruses produce several miRNAs and long noncoding RNAs (lncRNAs) that play important roles in regulating their survival (32–34). Bombyx mori cypovirus (BmCPV), a segmented double-stranded RNA virus, can form miRNAs, among which BmCPV-miR-3 can significantly promote BmCPV gene expression (32). The white spot syndrome virus (WSSV) can also encode miRNAs in early infection and promote WSSV replication in the host (33). Moreover, human adenoviruses can encode noncoding RNAs (ncRNAs) to promote viral proliferation (34). These studies suggested that the miRNAs and lncRNAs, both produced by viruses, can regulate the gene expression of the host and/or virus and influence the process of disease. It has been reported that HPV-encoded circE7 can promote the proliferation and growth of tumor cells (20). KSHV-encoded circRNAs can be encapsulated into KSHV particles, which may play an important role in the early stage of KSHV infection and the early immune response of host cells (22). Here, it was observed that the level of CyHV-2-encoded circ-udg increased with viral infection and that the overexpression of circ-udg promoted CyHV-2 gene expression. Altogether, these observations suggest that circ-udg may regulate viral infection.

It has been confirmed that circRNAs with IRES are capable of recruiting ribosomes for the translation of ORFs located downstream of IRES (16, 35, 36). It was also found that circRNAs with m6A methylation sites can initiate translation after they are methylated (17, 20). Various circRNAs have been reported to play important biological functions through encoded proteins (28, 37–40). However, there are only a few studies on virus-encoded circRNAs, and only circE7 derived from HPV has been reported to encode the E7 protein for the promotion of tumor cell proliferation and growth (20). Through bioinformatics analysis, we found that circ-udg has an IRES-like sequence. Experiments showed that circ-udg can encode a truncated UDG protein, circ-udg-P147, suggesting that the IRES on circ-udg has activity. Interestingly, the IRES-like sequence also contains the m6A site, raising the question that circ-udg may initiate the translation of circ-udg-P147 protein through the m6A site. Because the circRNA constructed *in vitro* cannot be m6A methylated (27), if circ-udg could only be translated through m6A, the circRNA synthesized *in vitro* should not translate circ-udg-P147 protein. However, we found that circ-udg synthesized *in vitro* could translate circ-udg-P147 protein. Therefore, we suggest that the IRES-like sequence of circ-udg can initiate translation. However, we cannot exclude that circ-udg formed in CyHV-2 infected cells can utilize both IRES and m6A to translate circ-udg-P147 protein.

UDG is an important DNA repair enzyme which is ubiquitous in organisms. It mainly excises the glycosidic bond of deoxyuracil on the DNA strand to form the “AP” site and then excises deoxyuracil through the joint action of other DNA repair enzymes to maintain stability of the genome (41–46). In addition to the host cells that can encode UDG, herpesvirus can also encode UDG (47–50). For example, the UDG protein encoded by herpes simplex virus 1 (HSV-1) can bind to the enzymes involved in HSV-1 replication, monitor for misincorporated deoxyuridines in DNA replication, and excise them in a timely manner to maintain genome stability, and HSV-1 encoded UDG can promote its own proliferation (51). Another herpesvirus, EBV, also encodes UDG, which contributes to viral replication (52). Genomic informatics analysis showed that CyHV-2 could also encode UDG. In this study, it was found that the *udg* gene can increase the expression of the CyHV-2 *ORF72* gene at both mRNA and protein levels. We speculate that circ-udg may regulate viral infection by controlling the level of UDG. Here, it was found that the circ-udg-P147 protein completely coincided with the C-terminal 147-amino-acid sequence of UDG protein, and both overexpression of circ-udg-P147 and UDG proteins could promote CyHV-2 gene expression. Also, circ-udg was found to promote the expression of CyHV-2 genes. However, the ability of circ-udg to promote the expression of CyHV-2 genes decreased after the start codon of circ-udg-P147 was modified to a stop codon. Therefore, it can be considered that circ-udg can promote the proliferation and replication of CyHV-2 by encoding circ-udg-P147.

Previous studies have reported that circ-SHPRH can encode a truncated sucrose nonfermentable-2 histone linker PHD RING helicase (SHPRH) protein that competitively binds to ubiquitinase, thus protecting the SHPRH protein from ubiquitination degradation (28). In this study, we observed that the transcript level of the *udg* gene was not affected by the level of circ-udg-P147 and that the protein level of UDG was increased in a circ-udg-P147 dose-dependent manner when circ-udg-P147 and UDG were coexpressed *in vitro*. Whether circ-udg-P147 also protects UDG from degradation in a manner similar to that of the truncated SHPRH protein needs to be further studied.

**FIG 8:**
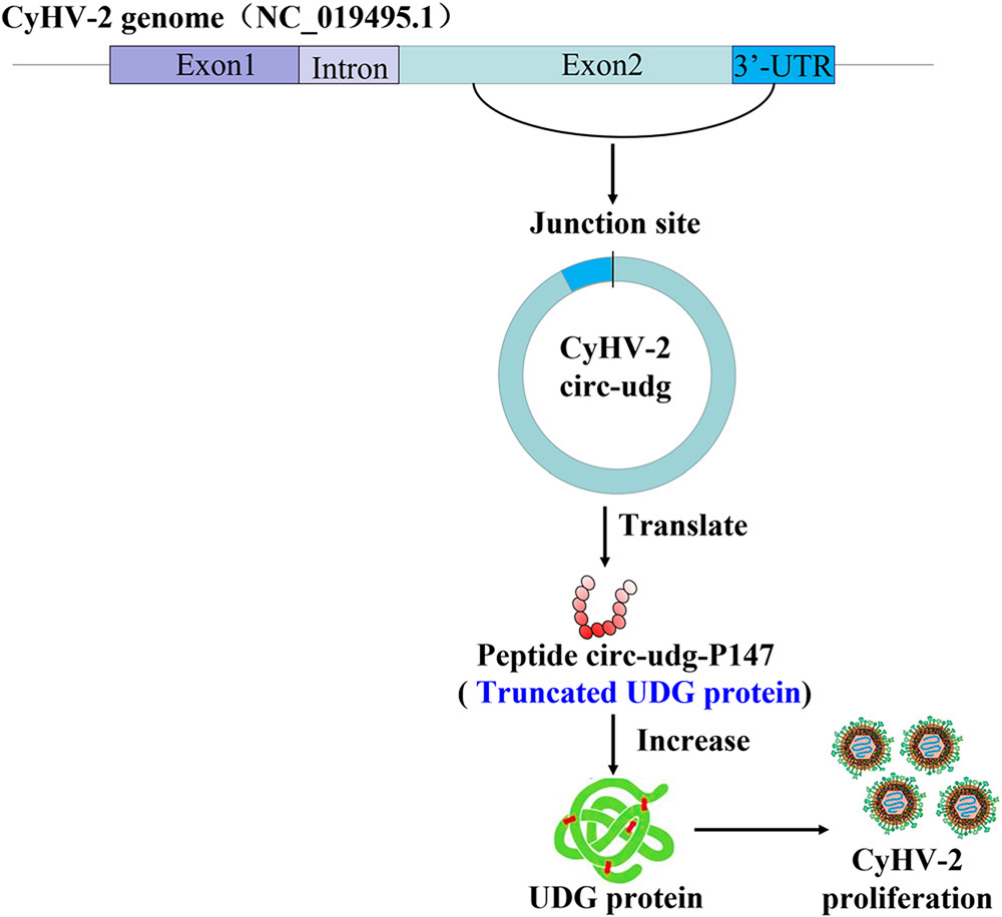
Circ-udg derived from CyHV-2 promotes viral replication. Circ-udg encodes a peptide circ-udg-P147 to increase UDG protein level, which, in turn, promotes CyHV-2 proliferation.

In conclusion, our study showed that CyHV-2 could encode circRNAs, of which circ-udg could promote the replication of CyHV-2. Furthermore, circ-udg was found to encode a truncated UDG protein, circ-udg-P147, which consists of 147-amino-acid residues. circ-udg-P147 could increase the level of UDG protein to promote CyHV-2 replication (Fig. 8). Our results deepen the understanding of the genetic information carried by the genome of CyHV-2 and provide new clues to explore the pathogenic mechanism of CyHV-2.

## MATERIALS AND METHODS

### Biological sample preparation.

The gibel carp infected with CyHV-2 were sampled from the aquaculture farm in Dafeng District, Yancheng City, Jiangsu Province, China.

The epithelioma papulosum cyprini (EPC) cells (kindly provided by Dong Qian, Ningbo University) were maintained in 199 medium (Sangon Biotech, Shanghai, China) containing 10% fetal calf serum (Gibco, Grand Island, NY, USA) at 26°C.

### CircRNA-Seq.

CircRNA-Seq was performed as described in our previous study (23). Briefly, seven tissues (kidney, heart, spleen, liver, gill, muscle, and intestine) from three CyHV-2-infected fishes were excised, and total RNA was extracted. The extracted RNAs were treated with RNase R (Epicentre, Madison, WI, USA) before the sequence libraries were constructed, according to the manufacturer’s protocol (Illumina, San Diego, CA, USA). Illumina sequencing was performed at Shanghai OE Biotech. Co., Ltd. The detailed processing of the raw data and analysis of the quality-controlled data were as described in our previous study (36). After quality control, the Bowtie2 tool (http://bowtie-bio.sourceforge.net/bowtie2/manual.shtml) was used to align the clean reads with the reference genome of CyHV-2 (GenBank accession number NC_019495.1) by the alignment of the anchors in the reverse orientation. CIRI2 (an efficient and unbiased algorithm for *de novo* circular RNA identification; version 2) was used to predict the circRNAs.

### Preparation of CyHV-2 stock.

The kidney and spleen tissues of CyHV-2-positive gibel carp were dissected and resuspended in sterile phosphate-buffered saline (PBS) at a ratio of 1 g of tissues to 20 mL of sterile PBS. The suspended tissues were ground in liquid nitrogen on ice, and the homogenate was centrifuged at 4000 × *g*/min for 30 min. Thereafter, the supernatant was collected and centrifuged at 8,000 × *g*/min for 30 min, and the resulting supernatant was the crude virus extract of CyHV-2. The copies of CyHV-2 in the crude extract were determined by real-time PCR, and the crude extract was stored at 4°C for subsequent experiments.

### Divergent PCR and Sanger sequencing.

Divergent PCR was used to confirm 10 circRNAs derived from CyHV-2. Divergent primers flanking the junction site and convergent primers targeting the junction site were designed based on the sequences of the circRNAs (Table 1). Total RNAs were extracted with the RNA Plus kit (TaKaRa, Dalian, China) according to the manufacturer’s instructions. After treatment with RNase R to remove the linear RNAs, the cDNAs were synthesized from 1 μg of total RNA with the First Strand cDNA synthesis kit (Transgene, Beijing, China) and random hexamers. The divergent primers and convergent primers were used to clone the junction site and circular sequence of each circRNA, respectively. The cDNA was amplified with PCR following the thermal cycling program of 95°C for 5 min, 94°C for 50 s, 30 cycles of annealing (at a temperature specific to the primer set used) for 50 s and extension at 72°C for 30 s, and a final extension at 72°C for 10 min. The PCR products were separated with 1% agarose gel electrophoresis. The recovered PCR products were cloned into pMD-19T vector (TaKaRa) for Sanger sequencing.

### Northern blotting.

Approximately 20 μg of total RNA and circular RNA was separated on a 1.2% agarose gel containing formaldehyde. After the RNA in the agarose gel was transferred to Hybond-N1 membranes (Roche, Basel, Switzerland), Northern blotting was performed with biotin-circ-udg, a biotin-labeled DNA oligonucleotide specific to circ-udg, in Church buffer (0.5 M NaPO_4_, 7% SDS, 1 mM EDTA, and 1% bovine serum albumin [BSA], pH 7.5) at 40°C and washed in 2× SSC (300 mM NaCl, 30 mM Na-citrate, pH 7.0) with 0.1% SDS at room temperature. The signals were detected with the biotin chromogenic detection kit (Thermo Scientific, Waltham, MA, USA). Biotin-circ-udg was synthesized by Sangon Biotech Co., Ltd. The sequence of biotin-circ-udg is listed in Table 1.

### Real-time PCR.

Reverse transcription for mRNA and circRNAs was performed using a murine leukemia virus reverse transcriptase (MLV-RT) kit (TransGen, Beijing, China) with random hexamers according to the manufacturer’s instructions. Real-time PCR was performed using a Bio-Rad PCR system (CFX96). Real-time PCR SYBR green mix (TransGen, Beijing, China) was employed with forward and reverse primers at 10 nM in a 20-μL reaction system. Relative expression levels were calculated using the threshold cycle (2^−ΔΔ^*^CT^*) method with the 40S rRNA gene as an internal control. To determine the absolute quantity of CyHV-2 DNA, the purified PCR product amplified from the genome of CyHV-2 with hel-1 and hel-2 primers was serially diluted and used as a template for real-time PCR to generate a standard curve. Primers used for real-time PCR are listed in Table 1.

### Construction of circRNA *in vitro*.

CircRNA was constructed according to our previous study (26). Briefly, the DNA sequences of circRNA containing the T7 promoter were amplified with a forward primer containing the T7 promoter and another specific primer. The purified PCR products were then used as the templates for *in vitro* transcription to obtain linear RNA by T7 RNA polymerase (TaKaRa). After that, the transcript was ligated with RNA ligase (NEB, Beijing, China), and the ligated product was treated with DNase I and RNase R to generate circRNA. The primer sequences for *in vitro* circularization of RNA are listed in Table 1.

### Prediction of circ-udg-encoded protein.

IRESite software (http://iresite.org/), ORF Finder software (https://www.ncbi.nlm.nih.gov/orffinder/), and N^6^-methyladenosine (m6A) sites online analysis software (http://www.cuilab.cn/sramp) were used to predict the internal ribosome entry site (IRES), open reading frame (ORF), and m6A methylation sites, respectively.

### Plasmids construction and transfection.

The linear full-length sequence of circ-udg was synthesized by GenScript (Nanjing, China), and the circ-udg expression vector was constructed by cloning the full-length sequence of circ-udg into the vector pIZT-LcR (26), with a nontarget pIZT-LcR vector as a negative control. To verify that ORF on circ-udg can encode proteins, a putative ORF encoding circ-udg-P147 was replaced with a fused DsRed with His tag to construct pIZT-LcR-circ-udg-DsRed. Meanwhile, an IRES site deletion vector, pIZT-LcR-circ-udg-DsRedmut, was also constructed. To explore the function of circ-udg-P147, a circ-udg-P147 expression vector was constructed. Briefly, using the pIZT-LcR-circ-udg plasmid as a template, the coding sequence of circ-udg-P147 was amplified by PCR and cloned into the pmCherry-C1 vector by EcoRI and BamHI to obtain the vector pmCherry-C1-circ-udg-P147. Additionally, the UDG coding sequence was cloned into the vector pZsCreen1-C1 by EcoRI and BamHI to construct a UDG expression vector, pZsGreen1-C1-udg. The plasmids were transfected using Lipofectamine 3000 (Invitrogen, Carlsbad, CA) according to the manufacturer’s instructions. Primers are listed in Table 1.

### Antibody generation.

To verify that circ-udg encodes circ-udg-P147, anti-circ-udg-P147 polyclonal antibody was prepared. Briefly, using pIZT-LcR-circ-udg plasmid as a template, the coding sequence of circ-udg-P147 was amplified by primers (circ-udg-P147-F and circ-udg-P147-R) and cloned into BamHI and XhoI sites of pET28a(+) vector. Then, pET28a-circ-udg-P147 was transformed into Escherichia coli BL21 strain and induced to express circ-udg-P147 protein by 0.5 mM IPTG (isopropyl-β-d-thiogalactopyranoside) at 37°C. The recombinant protein circ-udg-P147 was then purified by Ni-nitrilotriacetic acid (NTA) His Bind matrix column (Jinyitai, Wuhan, China). Finally, the purified proteins were analyzed by SDS-PAGE and Western blotting with the horseradish peroxidase (HRP)-labeled 6×His antibody and used to immunize mice every week for 4 weeks to prepare anti-circ-udg-P147 polyclonal antibody. The primer sequences used in this section are listed in Table 1.

### RNA interference.

The specific siRNAs for *udg* and circ-udg were synthesized by Sangon Biotech. They were transfected into EPC cells using Lipofectamine 3000 reagent (Thermo Fisher, Shanghai, China) according to the manufacturer’s protocol. A total of 100 pmol siRNAs mixed with 10 μL of liposome were added to 1 × 10^5^ cells. The cells were infected with CyHV-2 at 24 h posttransfection. The siRNA sequences used are listed in Table 2, and the silencing efficiency of siRNA is shown in Fig. S2 in the supplemental material.

**TABLE 2:** siRNAs sequences used in this study

Primer name	Sequence	
	Sense (5′→3′)	Antisense (5′→3′)
circ-udg-siRNA1	GGCUAAUUACCGAUUGUUUCA	AAACAAUCGGUAAUUAGCCUU
circ-udg-siRNA2	CCGAUUGUUUCAACGGUAUGG	AUACCGUUGAAACAAUCGGUA
circ-udg-siRNA3	ACCGAUUGUUUCAACGGUAUG	UACCGUUGAAACAAUCGGUAA
siRNA-NC	UUCUCCGAACGUGUCACGUTT	ACGUGACACGUUCGGAGAATT
udg-siRNA40	GGCUGUAGACCACGUUCAAAU	UUGAACGUGGUCUACAGCCGC
udg-siRNA244	GACCGACGAUGUGAUAAUACA	GACCGACGAUGUGAUAAUACA
udg-siRNA447	AGGUGUUGCUGUUGGGUAACA	UUACCCAACAGCAACACCUUG

### Cellular immunofluorescence.

First, the cells were fixed with 4% paraformaldehyde for 15 min at room temperature, followed by incubation with 0.1% Triton X-100 for 5 min. Then, cells were incubated with anti-CyHV-2-UDG antibody (1:500) at room temperature for 1 h. Cells were then washed three times with 0.01 M PBST (0.05% Tween 20 in PBS) and stained with fluorescein isothiocyanate (FITC)-labeled goat anti-mouse IgG (H+L) (1:300 dilution) (Servicebio, Wuhan, China) and DAPI (4′,6-diamidino-2-phenylindole; 1:1.000 dilution) (China, Beijing, Beyotime). Images were obtained using a fluorescence microscope (Leica, Wetzlar, Germany).

### Ethics approval and consent to participate.

The present study was approved by the Ethics Committee of Soochow University.

### Statistical analysis.

Unless otherwise indicated, statistical tests were conducted using Prism (GraphPad 6.0) software. Data are presented as mean ± standard error of the mean of the independent experiments.

### Data availability.

The sequencing data were deposited in the publicly accessible Sequence Read Archives of the National Center for Biotechnology Information (https://trace.ncbi.nlm.nih.gov/Traces/sra/?view=announcement) under the accession number SRR8293179.

Please contact the corresponding author for all data requests.
