# Supplementary material for: Circ-Udg Derived from Cyprinid Herpesvirus 2 Promotes Viral Replication
Source: Microbiol Spectr. 2022 Jun 30;10(4):e00943-22. doi: 10.1128/spectrum.00943-22 (PMC9431488; doi:10.1128/spectrum.00943-22)

Figure S1. Identification of circRNAs by divergent PCR.

A, Schematic diagram of the amplification of the circRNA junction sites by divergent PCR. The black arrows represent divergent primers. **B，** Sanger sequencing of the flanking sequence of circRNA junction sites.

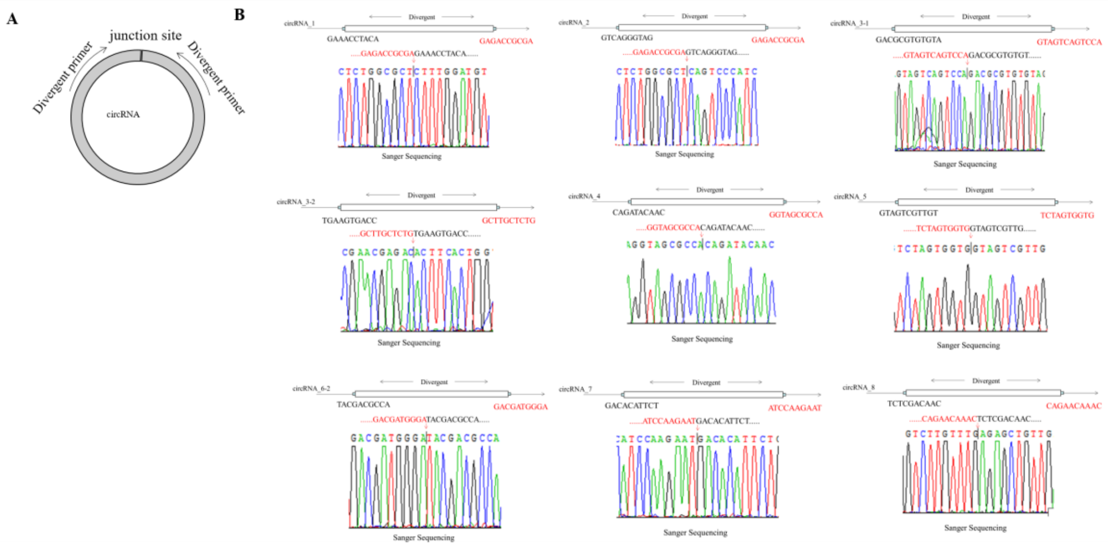

Figure S2 Silencing efficiency of siRNA.

A， Silencing efficiency of siRNA targeting circ-udg. the pIZT-LcR-circ-udg transfected cells (1×10^{6}) were transfected with 100 pmol circ-udg-siRNA1, circ-udg-siRNA2 and circ-udgsiRNA3, respectively, with circ-udg-siNC as negative control. After 48h, the expression of circ-udg was detected by qRT-PCR. **B,** The efficiency of silencing udg by udg-siRNAs(udg-siRNA40， udg-siRNA244 and udg-siRNA447). udg-siRNA40, udg-siRNA244 and udg-siRNA447 were transfected into cells (1×10^{6}). 48h later, the relative expression of udg was detected by real-time PCR.

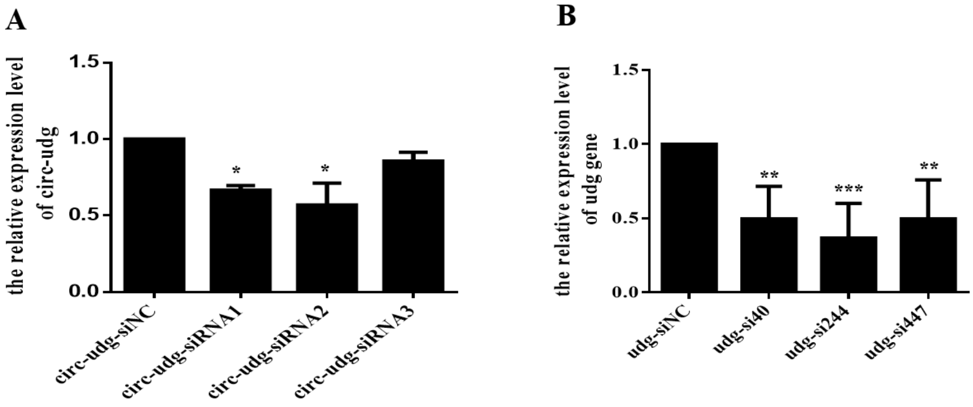

Supplement: Supplemental file 1 — Supplemental material. Download spectrum.00943-22-s0001.pdf, PDF file, 0.3 MB [file spectrum.00943-22-s0001.pdf]
